# Decoding Early Mycosis Fungoides: Histopathologic and Immunohistochemical Clues

**DOI:** 10.7759/cureus.57545

**Published:** 2024-04-03

**Authors:** Neslihan Kaya Terzi

**Affiliations:** 1 Pathology, Canakkale Onsekiz Mart University, Canakkale, TUR

**Keywords:** immunohistochemical markers, differential histopathological diagnosis, benign inflammatory dermatoses, early stage, mycosis fungoides

## Abstract

Introduction: Primary cutaneous lymphomas, notably mycosis fungoides (MF), present diagnostic challenges in recognizing early mycosis fungoides (eMF) due to their diverse clinical and histopathologic manifestations. The aim of our study was to use adjunctive histopathologic and immunohistochemical methods in eMF cases to make an early diagnosis and to facilitate differentiation from other dermatoses.

Methods: This retrospective study analyzed 35 cases of eMF diagnosed at a single center. Demographic and clinicopathologic data were collected, and histopathologic features were assessed. Comparative analyses were conducted with conditions mimicking eMF, including large plaque parapsoriasis (LPP), psoriasis, and chronic dermatitis. Immunohistochemistry for T-cell markers (CD3, CD4, CD8, CD2, CD7) was performed.

Results: With the scoring we applied in our study, a sensitivity of 91.43% (95% CI; 76.94% to 98.20%) and specificity of 85.71% (95% CI; 69.74% to 95.19%) for distinguishing eMF from LPP. Epidermotropism emerged as a crucial histopathologic marker, with a notable absence in most cases of cutaneous dermatitis (81.6% and 80% for CD and psoriasis, respectively) (P < 0.001). Immunohistochemistry revealed a T-helper phenotype (CD4+/CD8-) in the majority of eMF cases (78.1%), while CD4+/CD8+ and CD8+/CD4- patterns were less common (28.5% and 8.5%, respectively).

Conclusion: This study underscores the complexities in distinguishing eMF from inflammatory skin diseases, advocating for a comprehensive diagnostic approach.

## Introduction

Primary cutaneous lymphomas (PCLs) represent a heterogeneous category of non-Hodgkin's lymphomas characterized by an initial cutaneous manifestation without concurrent evidence of extracutaneous involvement upon diagnosis [1]. Mycosis fungoides (MF) stands out as the most prevalent subtype within this group. As an epitheliotropic mature T-cell lymphoma, MF is typified by small to medium-sized T cells exhibiting distinctive cerebriform nuclei [2]. The incidence of MF is notably higher in the elderly, with a mean age of onset ranging between 55 years and 60 years, and a male-to-female ratio of 1.6-2.0:1 [3].

Histopathologically, the lesions of MF present a range of features, progressing from confined epidermotropic, folliculotropic, and/or syringotropic lymphoid infiltrates in the initial phases to densely distributed dermal and subcutaneous infiltrates lacking prominent epidermotropism in the later stages of the disease [4,5]. Recognizing the early manifestations of eMF, such as patch or early plaque, poses a substantial diagnostic challenge. In eMF, atypical lymphocytes are organized along the dermal-epidermal junction and may display a perinuclear halo [4,5]. Distinguishing eMF from inflammatory skin conditions like large plaque parapsoriasis (LPP), psoriasis, and chronic dermatitis (CD) is often intricate. Clinical misinterpretation of eMF as LPP or CD frequently leads to delays in biopsy and histologic examination. The histological differential diagnosis is also intricate, necessitating sequential examinations and immunohistochemical testing for a definitive diagnosis. The necessary tips for dermatologic differentiation are as follows; MF begins as eczematoid or psoriasiform patches and plaques appearing in a "bath body" distribution, with most cases having a photoprotective "bath body" distribution involving the buttocks, trunk, and proximal limbs. In LPP, lesions are typically asymptomatic or accompanied by mild pruritus and are predominantly located on the trunk. It refers to a heterogeneous group of rare dermatoses, mostly occurring in older adults and characterized by erythematous and scaly patches of variable size, chronic course, and resistance to treatment. CD is characterized by localized areas of thickened, hyperkeratotic skin caused by friction or scratching. Psoriasis is a recurrent chronic disease affecting the skin, nails, and joints, usually characterized by well-circumscribed erythematous papules and silvery white scaly plaques [6]. The repetition of skin biopsies, often essential, contributes to increased patient morbidity and may be met with patient refusal on occasion [6,7].

Immunohistochemistry assumes a pivotal role in scrutinizing suspicious findings indicative of MF upon hematoxylin and eosin (H&E) staining of biopsy specimens.

In cases of MF, there is usually a notable prevalence of CD3+ cells, accompanied by a reduction in CD4+ cells and a decrease in one or more pan T-cell markers like CD2, CD5, and CD7. The identification of these characteristics in epidermal lymphocytes, as opposed to dermal lymphocytes, provides support for the diagnosis of eMF. Among the pan T-cell markers, CD7 is most commonly lost in MF, followed by CD5 and CD2 [8].

Despite the utilization of immunophenotyping and T-cell clonality studies, a definitive diagnosis of MF may remain elusive, necessitating vigilant clinical follow-up with repeat skin biopsies [8,9].

In our study, we aim to provide histopathologic and immunohistochemical clues to make an accurate and early diagnosis of eMF and to easily differentiate it from other dermatoses.

## Materials and methods

Patients

This research constitutes a single-center, retrospective study involving patients diagnosed with eMF at the Medical Pathology Laboratory of our institution.

A comprehensive search of the MF registry spanning January 2020 to June 2023 was conducted to identify all cases with a confirmed diagnosis of eMF. Inclusion criteria mandated both clinical and pathologic/immunohistochemical verification of eMF. Individuals showing signs of potential MF were not included in the study due to insufficient data in their medical records, unavailability of pathological specimens, and/or absence of follow-up records. The diagnosis in all cases was established through collaborative efforts involving a multidisciplinary team of dermatologists and pathologists, each possessing expertise in PCL. The diagnostic process relied on a fusion of clinical and pathologic/immunohistochemical examinations.

In addition, cases of diseases that histopathologically mimic eMF, specifically 60 CD, 35 LPP, and 20 psoriasis, were obtained from archival records and subjected to a comparative analysis.

Clinical and pathological features

Data pertaining to demographics, encompassing age and gender, as well as clinicopathologic characteristics such as the diagnosis date, age at diagnosis, duration between disease onset and diagnosis, and TNMB staging at the time of diagnosis, were obtained from both pathology and clinical records (Table 1). H&E-stained slides underwent reevaluation for predefined histopathologic features, categorized into epidermal and dermal features [10]. Epidermal features comprised parameters such as the intensity and pattern of epidermotropism, Pautrier's microabscesses, ulceration, spongiosis, folliculotropism, infiltrate depth and density, localization of atypical lymphocytes, presence and density of large cells, hyperkeratosis, parakeratosis, degree of epidermal thickness, and basal vacuolar damage. The dermal evaluation was performed according to inflammatory cell density, fibrosis, and the presence of eosinophils in the dermis [10,11].

The characteristic findings in CD are acanthosis and spongiosis in the epidermis and a mild infiltration of perivascular lymphocytes, histiocytes, and eosinophils in the upper dermis. Mild acanthosis, spongiosis, atypical lymphocytes in the epidermis, and superficial perivascular lymphocytic infiltration in the dermis are noted in LPP cases. Histopathologic features of psoriasis entailed specific characteristics such as psoriasiform epidermal hyperplasia, loss of the granular layer, Munro microabscesses, Kogoj pustules, and thinning of the suprapapillary area [12].

In cases of large plaque psoriasis, CD, and psoriasis, in addition to lymphocytic exocytosis, sometimes epidermatropism, atypical lymphocytic cells are present, and diagnosis is difficult because there is spongiosis or it is mild.

Immunohistochemical slides were removed from the archive and re-evaluated by an expert pathologist. Previously applied primary antibodies, including anti-CD3, anti-CD4, anti-CD8, CD2, and CD7 antibodies, underwent scrutiny [13]. Immunohistochemical CD3, CD4, and CD8 expression and CD4/CD8 ratio in atypical lymphoid cells were evaluated in all cases [7]. Expression of CD3, CD4, CD2, CD7, and CD8 was dichotomized as present or absent in two categories: no/little expression (≤25% positive cells) and (>25%) expression [10].

Patients were grouped according to the staging criteria of the International Society for Cutaneous Lymphomas and the European Organization for Research and Treatment of Cancer (EORTC), which are compatible with stage IA (T1N0M0), IB (T2N0M0), or IIA (T1 or 2N1 or 2M0) early-stage disease (ESD) [9].

Statistical analysis

For variables with a normal distribution, continuous data were expressed as the mean±standard deviation (SD). Two-group comparisons were conducted using the student's t-test and Mann-Whitney U test while the ANOVA t-test was employed for comparisons involving more than two groups. Exploratory analysis of qualitative clinical and histologic features was carried out using the chi-square test (χ^2^) or Fisher's exact test, depending on appropriateness.

Sensitivity, specificity, positive prognostic value, and negative prognostic value were computed for inter-entity comparisons.

All statistical analyses were executed using IBM SPSS Statistics for Windows, Version 23 (Released 2015; IBM Corp., Armonk, New York, United States), and a significance level was established at p < 0.05.

## Results

Demographic and disease-related characteristics

In the cohort of 35 individuals diagnosed with eMF, the mean age at the time of diagnosis was 63 years (range: 31-80), with a SD of 58.68±12.43 years. The male-to-female ratio was 1.5:1 among patients with eMF. Patches were observed in 20 cases (57.1%), while plaques were present in 15 cases (42.8%). Notably, although most of the lesions were situated in sunless areas, three cases (8.5%) were identified on the arm within sunlit regions.

In accordance with the most recent TNMB classification and staging systems, all 35 patients were classified as having ESD (≤IIA) at the time of diagnosis. This classification comprised 24 patients with stage IA, nine patients with stage IB, and two patients with stage IIA.

Table 1 provides a concise summary of the demographic and patient characteristics pertaining to individuals diagnosed with eMF. The average duration between the onset of lesions and the biopsy-based diagnosis of MF was 72 months (range: 6-96 months), while the meantime from disease onset to diagnosis was five months (range: 3-12 months).

**Table 1:** Demographic and clinical characteristics of 35 patients with mycosis fungoides

Characteristics	Values*
Age at time of diagnosis, years, median (range)	63 (31-80)
Age at time of diagnosis (years) (Mean SD)	58.68±12.43 (✝Mean±SD)
Sex (male/female), n (%)	
Male	21 (60)
Female	14 (40)
Clinical presentation	
Patch	20 (57.1)
Plaque	15 (42.8)
Lesion location	
Sun-exposed	3 (8.5)
Non-sun-exposed	32 (91.4)
Clinical stage at the time of diagnosis, n (%)	
IA	24 (68.5)
IB	9 (25.7)
IIA	2 (5.7)
T-stage at time of diagnosis, n (%)	
T1	25 (71.4)
T2	10 (28.5)
Nodal stage at the time of diagnosis, n(%)	
N1	1 (2.8)
N2	1 (2.8)
Mycosis fungoides variants, n (%)	
Classical	32 (91.4)
Folliculotropic	3 (8.5)
Time from onset of skin disease to initial mycosis fungoides diagnosis, years, mean (range)	0.51 (0.25-1)
Initial treatment, n (%)	
Topical steroids	30 (85.7)
Psoralen plus ultraviolet A	3 (8.5)
Ultraviolet B	1 (2.8)
No treatment	1 (2.8)
Follow-up time, years, mean (range)	6 (0.5-8)
Alive, n (%)	32 (91.4)
Abbreviations: *N (%); ✝: Mean±SD; SD: Standard deviation; T-stage: Tumor stage	

Clinical presentation

The predominant manifestation of MF in our study involved classic MF, observed in most cases (n = 32), constituting an incidence rate of 91.4%. A minority of patients, specifically three individuals (8.5%), were diagnosed with the folliculotropic variant of MF. Treatment modalities for these diagnoses included topical steroids in most cases (85.7%), with a smaller proportion receiving psoralen plus UV-A (8.5%) and UV-B (2.8%). Notably, a substantial proportion of patients, 32 individuals (91.4%), exhibited a favorable health status and maintained an ESD classification at the five-year follow-up assessment.

**Figure 1:**
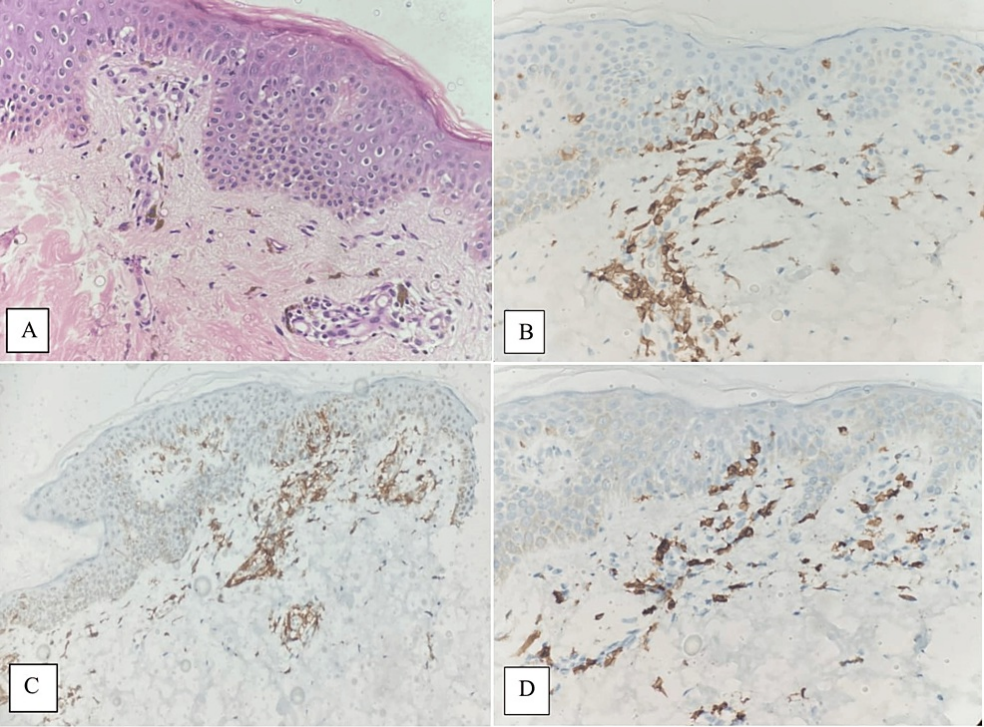
Microscopic photographs of a case diagnosed as eMF. Sparse atypical lymphocyte infiltration at the epidermis-dermis junction (H&E x400) (A). Membranous positive staining of atypical lymphocytes with immunohistochemical CD3 antibody (x400) (B). Membranous positive staining of atypical lymphocytes with immunohistochemical CD4 antibody (x200) (C). Less positive membranous staining in atypical lymphocytes in the epidermis with immunohistochemical CD8 antibody compared to CD4 antibody (x400) (D) eMF: Early mycosis fungoides; H&E: Hematoxylin and eosin

In two cases (5.7%), the clinical picture showed psoriasiform hyperplasia and focal granular layer loss, similar to psoriasis; however, histopathological analysis definitively identified these as cases of MF. In the retrospective evaluation of past biopsies from MF cases, only 24 cases had undergone a prior biopsy. Among these, two cases (8.3%) had initially received a diagnosis of LPP, and three cases (12.5%) were initially classified as lymphomatoid papulosis in another pathology department. Notably, the subsequent biopsies facilitated the accurate diagnosis of MF in these instances. Figure 1 shows a case of eMF with histopathologic and immunohistochemical features.

Other cases confused with eMF

**Figure 2:**
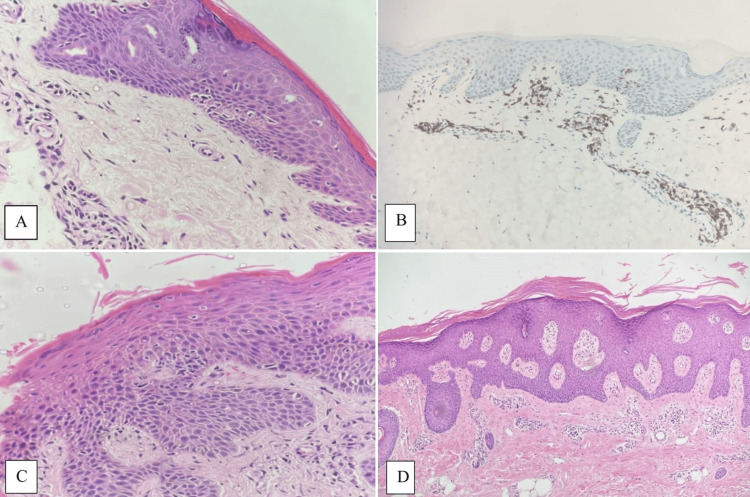
Microscopic photographs of PP, CD, and psoriasis, which can be histopathologically confused with eMF. Arrangement of atypical lymphocytes (H&E x400) in the basal epidermis of PP (A). Membranous positive staining with immunohistochemical CD3 antibody (x200) in sporadic T lymphocytes in the epidermis and more intense in the dermis (B). Epidermotropism with marked epidermal hyperplasia, hyperkeratosis, and spongiosis in a case of CD (H&E x400) (C). Psoriasis with psoriasiform epidermal hyperplasia, marked hyperkeratosis, and mild spongiosis (Hex100) (D) PP: Parapsoriasis; CD: Chronic dermatitis; eMF: Early mycosis fungoides

In our investigation, a total of 35 cases of LPP, 60 cases of CD, and 20 cases of psoriasis, all of which could potentially be confused with eMF, were included in the study. The histopathologic features of early non-MF dermatitis, encompassed within our study, were also extracted from archival records and subjected to comprehensive reevaluation. Various histopathologic features within the epidermal and dermal regions were meticulously assessed to identify specific diagnostic criteria. Table 2 and Figure 2 provide a detailed summary of the histopathologic features observed in all cases under investigation.

**Table 2:** Histopathological characteristics in the first available biopsy of 150 patients with MF, PP, CD, and psoriasis

Characteristics	MF (n,%)	PP (n,%)	CD (n,%)	Psoriasis (n,%)	P*
Epidermotropism					
None	0 (0)	5 (14.2)	49 (81.6)	16 (80)	<0.001
Minor	26 (74.2)	22 (62.8)	7 (11.6)	4 (20)	
Moderate	9 (25.7)	8 (22.8)	4 (6.6)	0	
Strong	0 (0)	0 (0)	0 (0)	0 (0)	
Pautrier’s microabscesses					
No	21 (60)	31 (88.5)	59 (98.3)	19 (95)	<0.001
Yes	14 (40)	4 (11.4)	1 (1.6)	1 (5)	
Ulceration					
No	34 (97.1)	27 (77.1)	50 (83.3)	17 (85)	0.11
Yes	1 (2.8)	8 (22.8)	10 (16.6)	3 (15)	
Spongiosis					
No	11 (31.4)	8 (22.8)	36 (60)	14 (70)	<0.001
Yes	24 (68.5)	27 (77.1)	24 (40)	6 (30)	
Folliculotropism					
No	8 (22.8)	35 (100)	58 (96.6)	20 (100)	<0.001
Yes	27 (77.1)	0 (0)	2 (3.3)	0 (0)	
Infiltrate density					
Low	31 (88.5)	26 (74.2)	16 (26.6)	9 (45)	<0.001
Moderate	4 (11.4)	9 (25.7)	12 (20)	11 (55)	
Dense	0 (0)	0 (0)	32 (53.3)	0 (0)	
Infiltrate depth					
Superficial	29 (82.8)	23 (65.7)	12 (20)	8 (40)	<0.001
Dermis	6 (17.1)	12 (34.2)	48 (80)	12 (60)	
Dermal infiltrate pattern					
Band like	21 (60)	7 (20)	12 12 (20)	3 (15)	<0.001
Perivascular	14 (40)	8 (22.8)	11 (18.3)	17 (85)	
Band and perivascular infiltrate pattern	0 (0)	20 (57.1)	37 (67.6)	0 (0)	
Atypical lymphocytes					
Only in epidermis	29 (82.8)	11 (31.4)	2 (3.3)	1 (5)	<0.001
Only in dermis	0 (0)	4 (11.4)	12 (20)	13 (65)	
Both in epidermis and dermis	6 (17.1)	20 (57.1)	46 (76.6)	6 (30)	
Large cells					
<10%	32 (91.4)	17 (48.5)	54 (90)	19 (95)	<0.001
10–50%	3 (8.5)	18 (51.4)	6 (10)	1 (5)	
>50%	0 (0)	0 (0)	0 (0)	0 (0)	
Eosinophils	1 (2.8)	14 (40)	36 (60)	6 (30)	<0.001
Papillary dermal fibroplasia	31 (88.5)	27 (77.1)	51 (85)	12 (60)	0.048
Corneal layer alterations					
Hyperkeratosis	28 (80)	30 (85.7)	52 (86.6)	19 (95)	0.29
Parakeratoses	21 (60)	24 (68.5)	48 (80)	20 (100)	
Epidermis					
Normal thickness	21 (60%)	24 (68.5%)	3 (5%)	0 (0%)	<0.001
Thinned	13 (37.1%)	2 (5.7%)	1 (1.6%)	0 (0%)	
Psoriasiform acanthosis	1 (2.8%)	5 (14.2%)	7 (11.6%)	18 (90%)	
Irregular acanthosis	1 (2.8%)	4 (11.4%)	49 (81.6%)	2 (10%)	
Vacuolar alteration of the basal layer	28 (80%)	15 (42.8%)	4 (6.6%)	0 (0%)	<0.001
Abbreviations: MF: Mycosis fungoides; PP: Parapsoriasis; CD: Chronic dermatitis. * Chi-square test (χ^2^) or Fisher's exact test					

All biopsies diagnosed with MF exhibited some degree of epidermotropism, with the majority displaying minimal epidermotropism in 26 out of 35 cases (74.2%). Conversely, most cases of LPP demonstrated an equivalent degree of epidermotropism in 22 out of 35 cases (62.8%), while the majority of CD and psoriasis cases exhibited no epidermotropism at all (81.6% and 80%, respectively) (P < 0.001). Notably, minimal epidermotropism was identified in seven cases (11.6%) and moderate epidermotropism in four cases (6.6%) of CD, with minimal epidermotropism also present in four cases (20%) of psoriasis. Epidermotropism in cases of psoriasis and CD is not considered a true epidermotropism because it is associated with spongiosis. Intense epidermotropism was not detected in any case across all biopsies.

Pautrier's microabscesses were observed in 14 out of 35 cases (40%) and superficial lymphoid infiltration in the epidermis in 29 out of 35 cases (82.8%) of MF. The occurrence of Pautrier microabscesses was notably lower in cases of LPP (11.4%), CD (1.6%), and psoriasis (5%) (P < 0.001). Conversely, superficial lymphoid infiltration in the epidermis was comparably high in MF cases (Table 2). Infiltration density was predominantly low in the majority of eMF (88.5%) and LPP (74.2%) cases, dense in most CD cases (53.3%), and moderate in the majority of psoriasis cases (55%) (P < 0.001).

The infiltrate in all primary biopsies predominantly comprised small to medium pleomorphic lymphocytes, with few or no larger cells (<10%). Notably, a higher proportion (10-50%) of infiltrates consisted of large cells in three out of 35 cases (8.5%) of MF, 18 out of 35 cases (51.4%) of LPP, six out of 60 cases (10%) of CD, and one out of 20 cases (5%) of psoriasis (P < 0.001).

Cases where many atypical lymphocytes were localized in the epidermis were diagnosed as eMF (82.8%). In cases of LPP (57.1%) and CD (76.6%), the predominant localization was observed in both the epidermis and dermis. Conversely, in psoriasis cases (65%), dermal localization was more prevalent (P < 0.001).

Follicular epitheliotropism/exocytosis was evident in 27 out of 35 cases diagnosed with MF. Notably, folliculotropism was absent in cases of LPP and psoriasis, whereas it was observed in only two cases (3.3%) of CD.

Histopathologic features such as hyperkeratosis, parakeratosis, spongiosis, and vacuolar degeneration of the basal layer in the epidermis were prevalent in most cases of eMF and LPP. CD and psoriasis cases similarly exhibited a high incidence of hyperkeratosis and parakeratosis. However, spongiosis was less frequently observed in CD and psoriasis cases compared to other diagnostic categories (40% and 30%, respectively). Notably, none of the psoriasis cases exhibited vacuolar degeneration of the basal layer, and the absence of ulcers was a consistent finding across almost all biopsies.

Epidermal thickness was predominantly normal in most cases diagnosed with eMF (60%) and LPP (68.5%), irregular acanthosis in the majority of CD cases (81.6%), and psoriasiform acanthosis in the majority of cases diagnosed with psoriasis (90%).

Upon evaluating the dermis, band-like infiltration was predominant in the majority of eMF cases (60%), while a combination of band and perivascular mixed infiltration was observed in the majority of LPP and CD cases (57.1% and 76.6%, respectively). A perivascular infiltrate was characteristic of most psoriasis cases (85%). Dermal fibroplasia was identified in most of all biopsies (60-85%), with a particularly high prevalence in MF cases (88.5%). Eosinophils in the dermis were primarily observed in cases with LPP (40%) and CD (60%), while eosinophil infiltration was present in only 2.8% of MF cases.

Upon applying the scoring system recommended for eMF, 32 cases (91.4%) achieved a score of four and above without the necessity for immunohistochemical markers. In three cases (8.5%), the diagnosis was confirmed through immunohistochemical studies. The scoring system was also applied to cases that could potentially be confused with MF. Upon performing clinical and pathologic scoring of cases diagnosed with LPP, the score remained three in 30 cases (85.7%). In five cases (14.2%), the score was four, and the diagnosis of MF was excluded through immunohistochemical examination. The sensitivity of the scoring system for differentiating eMF and LPP was determined to be 91.43% (95% CI; 76.94% to 98.20%), with a specificity of 85.71% (95% CI; 69.74% to 95.19%), positive predictive value of 99.18% (95% CI; 98.17% to 99.64%), and negative predictive value of 34.48% (95% CI; 15.03% to 61.03%).

Immunohistochemical examination

**Figure 3:**
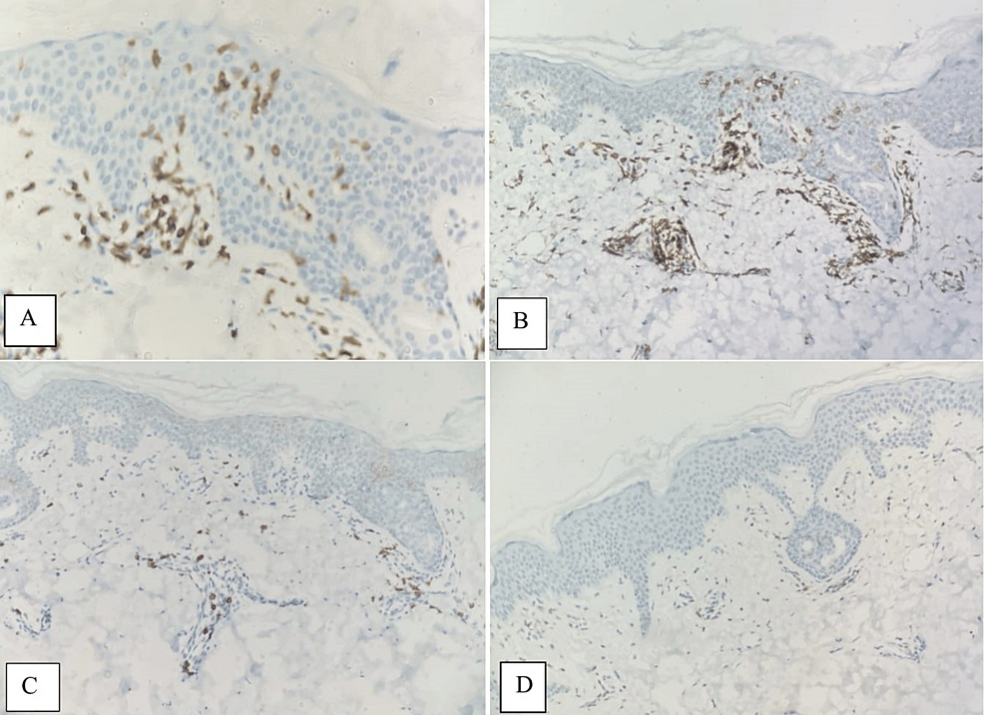
Immunohistochemical examination of an eMF case. Membranous staining with CD3 antibody in the epidermis is consistent with the presence of atypical lymphocytes suggestive of epidermotrpism (x400) (A). Membranous positive staining of atypical lymphocytes in the epidermis with CD4 antibody (x200) (B). No staining was observed in atypical lymphocytes in the epidermis with CD8 antibody (x200) (C). CD2 antibody showed loss of expression in atypical lymphocytes (x200) (D) eMF: Early mycosis fungoides

The findings of the immunohistochemical examination conducted on cases diagnosed with eMF are presented in Table 3. Immunohistochemical staining revealed a T-helper phenotype characterized by CD4+/CD8- in 25 out of 35 cases (71.4%) that were subjected to testing (Figure 3). Seven cases exhibited dual positivity for CD4 and CD8, representing 20% of the examined cases. CD8+/CD4- was detected in three cases, constituting a rate of 8.5%.

**Table 3:** Immunohistochemical results of the mycosis fungoides, n(%)

	No/low exp.	Mild-severe exp.	P*
CD3	1 (2.8)	34 (97.1)	<0.001
CD4	3 (8.5)	32 (91.4)	
CD8	25 (71.4)	10 (28.5)	
CD2	28 (80)	7 (20)	
CD7	30 (85.7)	5 (14.2)	
Abbreviations: exp: expression; *chi-square test.			

The pan T cell marker CD3 was preserved in 34 out of 35 cases tested, accounting for a preservation rate of 97.1%; however, partial loss of CD2+ and CD7+ was observed in 28 out of 35 cases (80%) and 30 out of 35 cases (85.7%), respectively.

Intense staining was observed with the CD3 antibody in most cases (74.2%), with only one case (2.8%) exhibiting a very low expression level, deemed as negative.

## Discussion

The average age at the time of diagnosis among our cohort of patients afflicted with MF was determined to be 63 years, marginally surpassing the figures documented in existing literature [4,10]. The observed preponderance of male cases aligns with the findings of prior investigations [10,14,15].

Clinical presentations predominantly featured an erythematous patch succeeded by a plaque, a trend consistent with previous studies by Khader et al., which identified plaque as the most prevalent lesion, either in isolation or in combination [16]. This pattern is further corroborated by the work of Eklund et al., who similarly identified plaque as the predominant lesion [17]. In contrast, a study conducted by Arafah et al. among Saudi patients identified a patch as the most frequently occurring lesion [18].

The classical subtype of MF constituted most cases (91.4%), with the folliculotropic MF (FMF) variant comprising a minor proportion (8.5%). This distribution is congruent with comparable studies underscoring that FMF constitutes 10% or less of all MF cases [4,19].

The temporal gap between the onset of lesions and the diagnostic biopsy for MF was determined to be 72 months on average, slightly shorter than reported in a Danish study [10].

Over the follow-up duration, progression to the subsequent disease stage manifested in only three patients (8.5%), a notably lower incidence compared to studies reporting higher transition rates, approximately 30-35% [20]. In a comprehensive US study, a mere 11.6% of patients advanced to a more severe disease stage [15].

The predominant initial approach to patients presenting with dermatitis-like symptoms involved the administration of topical steroids, with biopsy acquisition ensuing in cases of treatment failure [20,21]. Notably, the use of topical steroids, while efficacious in alleviating inflammation, posed challenges in histologic interpretation during the eMF [21]. Consistent with the EORTC consensus guidelines for eMF treatment, topical steroids alone constituted the primary therapeutic modality in 85.7% of cases within our cohort [21]. Furthermore, only four patients received psoralen ultraviolet A (PUVA)-A and PUVA-B as first-line treatment, indicating a limited utilization of these modalities in our study population.

Differentiation with other dermatitis

Within our study cohort, the prevalent histopathologic diagnoses preceding the identification of MF were non-specific dermatitis and atypical lymphocytic infiltration. Specifically, 8.3% of patients exhibited LPP, while 12.5% displayed lymphomatoid papulosis in biopsies antedating the MF diagnosis. Extant research indicates that LPP harbors the potential for transformation into MF [22], and lymphomatoid papulosis is correlated with an elevated risk of MF development [1,23].

Distinguishing MF from LPP presents a formidable challenge [7]. LPP, acknowledged to progress to T-cell lymphoma, including MF, in a subset of cases (7.5-14% based on diverse studies) [24], lacks clear differentiation at clinical, histologic, and molecular levels [7]. Varied perspectives position LPP either as a patchy stage of MF or as a benign condition with a propensity for transformation into PCL due to chronic antigen stimulation, akin to CD [25]. Histologically, LPP is characterized by superficial lymphocytic infiltration, with over 30% of cases progressing to MF [25,26]. The ambiguity associated with LPP stems from its non-specific clinical presentation, unpredictable evolution, and challenging histologic diagnosis [25].

Early MF often exhibits interface changes mimicking other inflammatory dermatoses, leading to delayed diagnosis and misclassification [14,27]. Repeated biopsies and robust clinicopathologic correlation, as well as careful evaluation of histopathologic clues and immunohistochemistry for CD4, CD8, CD2, and CD7, and TCR clonality tests are essential for definitive diagnosis [28].

In our study, cases resembling psoriasis clinically but lacking a personal history of the disease manifested in only 5.7% of instances. Given the prevalent use of immunosuppressive/immunomodulatory agents for psoriasis, vigilance is warranted for potential exacerbation or aggressive behavior of underlying MF. Consequently, in psoriatic cases unresponsive to treatment or exhibiting progression despite therapy, clinical reassessment for concurrent or misdiagnosed MF is imperative [4].

Histopathologic features distinguishing MF cases encompassed epidermotropism, Pautrier microabscesses, folliculotropism, vacuolar degeneration in the basal layer, and papillary dermal fibroplasia [24]. Unexpectedly, spongiosis, observed in 68.5% of MF cases, challenges the conventional expectation of its absence in MF. Similarly, spongiosis featured prominently in LPP (77.1% of cases), undermining its utility as a distinctive epidermotropism marker. Notably, the epidermis of MF cases predominantly housed atypical lymphocytes (82.8%), distinguishing them from LPP and contact dermatitis, where both epidermal and dermal localization of atypical lymphocytes was more prevalent (57.1% and 76.6%, respectively).

The diagnostic utility of the eMF scoring system proposed by Pimpinelli et al. revealed its efficacy in diagnosing MF without the necessity for immunohistochemistry in most cases (91.43% sensitivity, 85.71% specificity) [11]. Nevertheless, the lack of immunohistochemical studies and TCR clonality analysis in some cases constitutes a study limitation. Further refinement of the algorithm may enhance its specificity and sensitivity.

A retrospective analysis by Amorim et al. underscored the significance of combining clinical, pathologic, and immunohistochemical criteria, achieving a 91% diagnostic success rate for eMF [9]. Despite the algorithm's high sensitivity, ongoing research may be warranted to enhance its specificity.

It is imperative to acknowledge the limitations of our study, primarily its retrospective nature, reliance on a limited geographical cohort, and the ensuing challenge of generalizing findings to broader populations.

## Conclusions

These results accentuate the complexities encountered by clinicians and pathologists in the diagnosis of eMF, given its heterogeneous presentation and potential to mimic various inflammatory skin diseases. The implications include delayed diagnoses, deferred therapeutic interventions, and a call for future investigations into diagnostic and prognostic biomarkers in the realm of eMF.
